# Short versus standard implants at sinus augmented sites: a systematic review and meta-analysis

**DOI:** 10.1007/s00784-022-04628-1

**Published:** 2022-09-07

**Authors:** Manuel Toledano, Enrique Fernández-Romero, Cristina Vallecillo, Raquel Toledano, María T. Osorio, Marta Vallecillo-Rivas

**Affiliations:** Faculty of Dentistry, University of Granada, Colegio Máximo de Cartijo S/N, 18071 Granada, Spain

**Keywords:** Short, Implants, Survival, Bone, Loss, Review

## Abstract

**Background:**

Short implants are proposed as a less invasive alternative with fewer complications than standard implants in combination with sinus lift. The aim of this systematic review and meta-analysis was to state the efficacy of placing short implants (≤ 6 mm) compared to standard-length implants (≥ 8 mm) performing sinus lift techniques in patients with edentulous posterior atrophic jaws. Efficacy will be evaluated through analyzing implant survival (IS) and maintenance of peri-implant bone (MBL).

**Methods:**

Screening process was done using the National Library of Medicine (MEDLINE by PubMed), EMBASE, the Cochrane Oral Health, and Web of Science (WOS). The articles included were randomized controlled trials. Risk of bias was evaluated according to The Cochrane Collaboration’s tool. Weighted means were calculated. Heterogeneity was determined using Higgins (*I*^2^). A random-effects model was applied. Secondary outcomes such as surgical time, patient satisfaction, mucositis and peri-implantitis, pain, and swelling were analyzed.

**Results:**

Fourteen studies (597 patients and 901 implants) were evaluated. IS was 1.02 risk ratio, ranging from 1.00 to 1.05 (CI 95%) (*p* = 0.09), suggesting that IS was similar when both techniques were used. MBL was higher in patients with standard-length implants plus sinus lift elevation (*p* = 0.03). MBL was 0.11 (0.01–0.20) mm (*p* = 0.03) and 0.23 (0.07–0.39) mm (*p* = 0.005) before and after 1 year of follow-up, respectively, indicating that the marginal bone loss is greater for standard-length implants.

**Discussion:**

Within the limitations of the present study, as relatively small sample size, short dental implants can be used as an alternative to standard-length implants plus sinus elevation in cases of atrophic posterior maxilla. Higher MBL was observed in the groups where standard-length implants were used, but implant survival was similar in both groups. Moreover, with short implants, it was observed a reduced postoperative discomfort, minimal invasiveness, shorter treatment time, and reduced costs.

**Clinical Clinical relevance:**

The low MBL promoted by short implants does contribute to a paradigm shift from sinus grafting with long implants to short implants. Further high-quality long-term studies are required to confirm these findings.

## Introduction

Postextraction alveolar ridge remodeling frequently results in reduced bone dimension or even in severe ridge atrophy [1], coupled with age-liked sinus pneumatization [2] that usually compromises bone height. Maxillaris sinus floor elevation has become the most reliable, commonly used procedure to increase bone height in the posterior maxilla [2, 3].

Patients with extremely atrophic posterior maxillae not only require for rehabilitation with fixed prostheses, dental implants after sinus lift procedures. They also, very often, are in need of zygomatic implants and sometimes titled or pterygoid implants [4]. In particular, the posterior maxilla is a challenging area for dental implants’ placement [1, 2]. Complications, such as postoperative sinusitis, partial, or total graft failure, may occur after sinus floor elevation, appearing up to 38% of patients, and implants fail in up to 17% of patients within 3 years [2]. Augmentation procedures may, even more, require hospitalization and longer times (up to 1 year) for rehabilitating the patients [5]. Therefore, evidence of these complications together with the increasing predictability of less invasive alternatives for implant placement might reserve the use of sinus floor elevation procedures only for cases of minimum height of alveolar bone [6, 7].

The placement of short dental implants instead of sinus floor elevation technique in atrophic posterior maxillae (6 mm ≤ residual bone height < 8 mm), without jeopardizing stability, has been a long-time waiting hope in dental implantology [8]. The application of such short implants could eliminate the need of sinus floor elevation and reduce the associated complications, treatment time, and cost, while increasing patients’ acceptance [1]. A short implant is an implant with its designed intrabony length < 8 mm [8]. Short dental implants are currently used, besides, as an alternative to longer implants in purposely augmented bone, in case of reduced bone volume [9], to support fixed prosthesis in the rehabilitation of atrophic jaws [10]. In addition, narrow and short implants can be used as an alternative to longer and wider implants in augmented zones with reduced bone volume [11, 12]. In cases of suspected graft infection, moreover, it may be wiser to remove the graft completely and use short implants instead [5].

Questions were raised, however, whether shorter dental implants might replace sinus elevation procedures in conjunction with longer dental implants. While longer implants might have a better long-term prognosis in non-augmented bone, the long-term prognosis of short implants compared to longer implants placed in augmented bone is still unknown [5]. The most frequently reported criteria for implant success are based on the implant level, i.e., survival rates (IS) and marginal bone loss (MBL) [13]. Implant survival is defined as the implant remaining in situ at the follow-up examination [14]. The marginal bone loss is measured by the radiographic bone level, i.e., the distance between the implant shoulder and the bottom of the defect at bone-level implants [15].

Short implants could be a simpler, cheaper, less invasive, and faster alternative if they could provide similar clinical outcomes to longer implants placed in augmented bone [16]. Despite the tendency for increased early failure of short implants in smokers, machined surface implants, and severe reabsorbed posterior maxilla [17, 18], it has been previously reported that no statistically significant differences in IS or MBL were found after placement of ≤ 8 mm implants compared with standard-length implants > 8 mm, after 3 years of functional implant loading [19]. Even more, in another systematic review [20], short (< 6 mm) and longer implants (> 10 mm) with sinus floor elevation were compared and analyzed. A total of seven randomized controlled clinical trials (RCTs) involving 310 patients were included. The follow-up reached more than 3 years for several studies. Authors declared that no significant differences with regard to MBL and IS rate were found between each group at each time of the follow-up, 1 up to 3 years and more than 3 years.

No consensus has been reached on the controversial issue that whether the length of implants is considered as short or standard implant. According with the last European Association of Dental Implantologists consensus in 2016, ultrashort implants are defined as < 6 mm and dental implants with length of 8 mm or more (≥ 8 mm) could be accepted as standard-length implants [1, 21]*.* Pending more long-term studies, the success rates of short implants in the posterior maxillae are still controversial [22]. The aim of this systematic review was, therefore, to address the following focused question: In patients with edentulous posterior atrophic jaws, what is the efficacy of placing short implants (≤ 6 mm) compared to standard-length implants (≥ 8 mm) performing sinus lift techniques, in terms of implant survival and maintenance of peri-implant bone?

## Material and methods

### Protocol and registration

The study protocol of the present systematic review and meta-analysis was prepared following the model propose in the PRISMA statement and looking for the greatest transparency structured according to the PRISMA checklist [23]. The developed protocol was previously registered and allocated with the registration number 295642 in the International Prospective Register of Systematic Reviews (PROSPERO).

### Focused question

This review intends to answer the following focused query designed in accordance with the PICO question [24]: In patients with edentulous posterior atrophic jaws, what is the efficacy of placing short implants (≤ 6 mm) compared to standard-length implants (≥ 8 mm) performing sinus lift techniques, in terms of implant survival and maintenance of peri-implant bone?

The PICOs elements were as follows:

- *Population (P)*: Patients not affected by systemic conditions, older than 18 years, with edentulous posterior atrophic jaws requiring implant rehabilitation.
- *Intervention (I)*: Implant rehabilitation with extra-short and short implants (≤ 6 mm).
- *Comparison (C)*: Implant rehabilitation with standard implants (≥ 8 mm) associated with maxillary sinus elevation.
- *Outcome (O)*: Outcomes measuring survival rate of the implants (implants lost during study follow-up), and mean differences of marginal bone loss as primary outcomes and secondary variables such as implant characteristics, implant stability, periodontal health parameters, and patient-reported outcome.
- *Study (S)*: Randomized controlled clinical trials.

### Search strategy

An electronic search across the National Library of Medicine (MEDLINE by PubMed), the Cochrane Oral Health Group Trials Register, EMBASE, and Web of Science (WOS) was performed for clinical studies. Only studies published in English between 1993 and February 2022 were considered. Reference lists of the previous reviews and included studies were screened trying to search for relevant manuscripts that were missing after the electronic screening. Bibliographies of eligible articles were manually searched.

The search strategy included the following word combinations: ((ultra-short dental implant) OR (extra short dental implant) OR (short dental implant) OR (< 6-mm dental implant) OR (5-mm dental implant) OR (4-mm dental implant)) AND ((atrophic posterior maxilla) OR (sinus lift) OR (sinus floor elevation) OR (sinus membrane elevation) OR (sinus floor augmentation).

### Eligibility: inclusion and exclusion criteria for studies

In order to increase the quality, the following inclusion criteria have been chosen:

1. Randomized controlled clinical trials.
2. Comparisons between short implants (≤ 6 mm) without maxillary sinus augmentation and standard-length implants (≥ 8 mm) with maxillary sinus augmentation in the same study.
3. Studies that consider short implants, those with a length equal or less than 6 mm.

Studies meeting at least one of the following criteria were excluded:

1. In vitro and pre-clinical studies, case series or case reports, retrospective studies, systematic reviews.
2. Full-text publications not available in English language.
3. Studies with less than 6 months of follow-up.
4. Studies that consider as short implants those with more than 6 mm of length.

### Study selection and data extraction

Two authors (EF, CV) independently screened the titles and abstracts derived from the online search considering the inclusion and exclusion criteria. The complete articles sourced via eligible titles and abstracts were obtained and examined independently to determine eligibility. Disagreements between these reviewers related to the selection and inclusion of any specific paper were discussed until either a consensus was reached, or a third reviewer (MT) led to an agreement and determined inclusion or exclusion. All reports excluded at this stage were formally recorded, as well as the reason/s for their exclusion. Cohen’s kappa coefficient was calculated as a measure of agreement between the two readers.

Two investigators (EF and CV), independently, extracted the data from included articles and assessed the risk of bias in duplicate and thereafter discussed to find an agreement. In case of disagreement, the judgment of a third reviewer (MT) was decisive. Data extracted were the following: (1) authors and year of publication; (2) number of patients and implants; (3) follow-up periods; (4) implant treatment modality; (5) implant survival; (6) marginal bone loss; (7) summary results; (8) sinus lift surgery; and (9) type of restoration. To complete the search, information regarding secondary outcomes [diameter, implant stability quotient (ISQ), buccal bone thickness (BBT), bleeding on probing (BoP), probing depth (PD), surgical time (ST), patient satisfaction, peri-implantitis/mucositis, pain/swelling, and complications] were also reported.

### Assessment of risk of bias

Methodological quality and risk of bias were evaluated by two reviewers according to the Cochrane Collaboration’s tool [25]. The assessment criteria were separately prepared for different domains. For each domain, the risk of bias was graded as high, low, or unclear, and studies were classified as “High risk,” “Some concerns,” or “Low risk.” When there was a major disagreement, a third reviewer participated in the discussion until a consensus was reached.

### Data analyses

For the primary outcomes, implant survival (in terms of number of implants that exceed the follow-up periods), and marginal bone loss [in terms of MBL (mm)], descriptive statistics were used. For MBL, weighted means (CI 95%) were calculated, including total sample size, inverse variance, and standard error of the treatment effect. For IS, risk ratio (RR) (CI 95%) was assessed using chi-square test [Mantel–Haenszel (M-H)]. Due to the clinical heterogeneity detected between studies, a random-effects model was applied, in order to analyze effect sizes. For MBL analysis, two subgroups were established. Hence, comparisons were performed between the experimental and control groups considering the time of follow-up (≤ 1 year, > 1 year). Data were analyzed with RevMan 5.4 (The Cochrane Collaboration, Oxford, UK). Statistical significance was set at 0.05.

### Risk of bias across studies

The variation across the included studies, or heterogeneity, was determined using Higgins (*I*^2^). Funnel plot was produced by RevMan 5.4 to represent systematic heterogeneity and publication bias.

## Results

### Search results

**Fig. 1:**
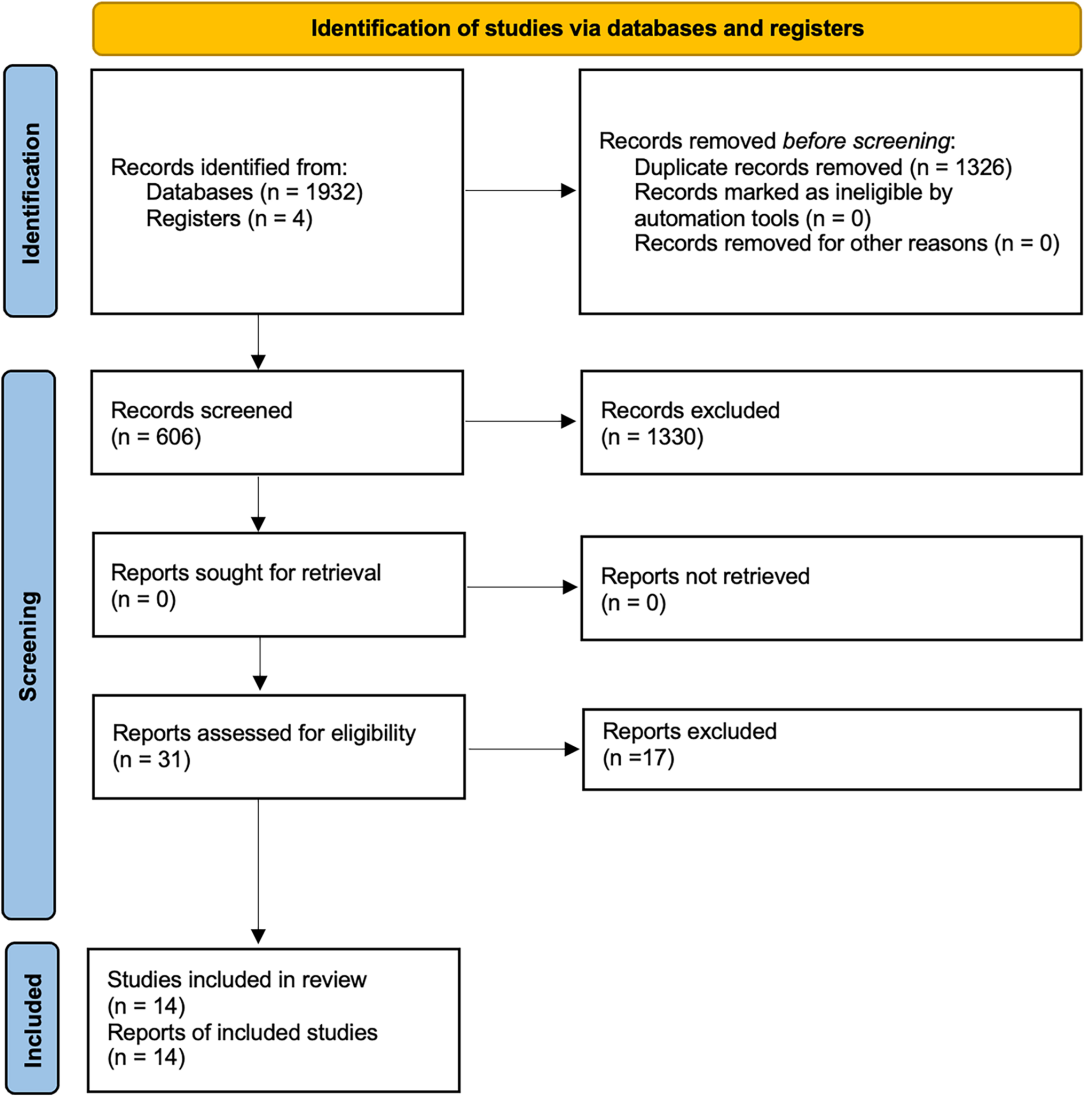
PRISMA flow diagram for studies inclusion process

Search results based on the PRISMA guidelines are presented in Fig. 1. The electronic and manual searches yielded 1932 references in total (PubMed: 602; EMBASE: 587; Cochrane Library: 126; WOS: 617; manual search in other sources: 4). Subsequent to duplicate removal and after reading of titles and/or abstracts, 31 articles were selected. Then the full text of all the selected articles was reviewed for the inclusion criteria. Following the evaluation and deep read of articles, 17 were excluded. Therefore, 18 articles were included in the final selection and reserved for data extraction. The reasons for exclusion of articles from the study were as follows: 3 studies defined implants > 6 mm in length as short implants, and 14 studies presented patients or data repeated in other articles included (Table 1). The inter-reviewer agreement in the screening and inclusion process corresponded to 0.95 with de Cohen’s kappa for assessment of the title and abstract, and full-text evaluation. The extracted data for each reviewed article are shown in Table 2.

**Table 1:** Excluded studies and reason for exclusion

Article	Reason for exclusion
Pohl 2017 [26], Esposito 2016 [27], Zhang 2017 [8], Shi 2015 [28], Esposito 2014 [29], Guljé 2014 [30], Pistilli 2013a, 2013b [31, 32], Felice 2013 [33], Felice 2012 [34], Esposito 2012 [35], Felice 2011 [36], Esposito 2011 [37], Felice 2009 [38]	Studies that correspond to the shorter or longer follow-up of an included study
Taschieri 2018 [39], Esposito 2015 [40], Thoma 2015 [41]	Studies that consider as short implants those with more than 6 mm of length

**Table 2:** General overview of the included studies

Nº	Author	Follow-up	Group	Treatment modality	Implant survival (%)	Marginal bone loss *n (m* ± *SD)*	Summary results	Sinus surgery and restoration
1	Magdy et al. 2021 [1]	1 year	Test (*n* = 24 I)	Short implants (5.5 mm)	87.5% (3 failed) (*n* = 24)	1 yr: 21 (0.91 ± 0.69)	IS: No SS results MBL: SS results (< short implants)	OSFE SC
			Control (*n* = 24 I)	SFE + Standard-length implants (10 mm)	95.8% (1 failed) (*n* = 24)	1 yr: 23 (1.44 ± 0.72)		
2	Shi et al. 2021 [42]	3 years	Test (*n* = 67 I)	Short implants (6 mm)	91.8% (6 failed) (*n* = 67)	3 yr: 61 (0.53 ± 0.35)	IS: SS results (< short implants) MBL: No SS results	OSFE SC
			Control (*n* = 62 I)	OSFE + Standard-length implants (8 mm)	97.08% (1 failed) (*n* = 62)	3 yr: 61 (0.50 ± 0.30)		
			Control (*n* = 70 I)	OSFE + Standard-length implants (10 mm)	100% (0 failed) (*n* = 70)	3 yr: 70 (0.53 ± 0.28)		
3	Rossi et al. 2021 [43]	2 years	Test (*n* = 12 I)	Short implants (4 mm)	100% (0 failed) (*n* = 12)	1 yr: 12 (0.21 ± 0.35) 2 yr: 12 (0.44 ± 0.37)	IS: No SS results MBL: No SS results	LWT + Graft + RCM FPDs
			Control (*n* = 10 I)	SFE + Standard-length implants (10 mm)	100% (0 failed) (*n* = 10)	1 yr: 10 (0.58 ± 0.44) 2 yr: 10 (0.84 ± 0.68)		
4	Nielsen et al. 2021 [3]	1 year	Test (*n* = 20 I)	Short implants (6 mm)	100% (0 failed) (*n* = 20)	1 yr: 20 (0.60 ± 0.17)	IS: No SS results MBL: No SS results	LWT + Graft + RCM SC
			Control (*n* = 17 I)	SFE + Standard-length implants (13 mm)	100% (0 failed) (*n* = 17)	1 yr: 17 (0.51 ± 0.14)		
5	Esposito et al. 2019 [10]	5 years	Test (*n* = 36 I)	Short implants (5 mm)	97.2% (1 failed) (*n* = 36)	1 yr: 36 (1.16 ± 0.3) 5 yr: 35 (1.58 ± 0.38)	IS: No SS results MBL: SS results (< short implants)	LWT + Graft + RCM SC and FPD
			Control (*n* = 37 I)	SFE + Standard-length implants (≤ 10 mm)	100% (0 failed) (*n* = 37)	1 yr: 37 (1.53 ± 0.59) 5 yr: 37 (2.11 ± 0.66)		
6	Felice et al. 2019 [5]	5 years	Test (*n* = 39 I)	Short implants (6 mm)	95.5% (2 failed) (*n* = 39)	1 yr: 39 (1.41 ± 0.31) 5 yr: 37 (1.93 ± 0.54)	IS: No SS results MBL: SS results (< short implants)	LWT + Graft + RCM SC and FPD
			Control (*n* = 44 I)	SFE + Standard-length implants (≤ 10 mm)	100% (0 failed) (*n* = 44)	1 yr: 44 (1.53 ± 0.29) 5 yr: 44 (2.28 ± 0.46)		
7	Felice et al. 2019 [44]	5 years	Test (*n* = 34 I)	Short implants (5 mm)	91.2% (3 failed) (*n* = 34)	1 yr: 34 (1.06 ± 0.53) 5 yr: 31 (1.65 ± 0.63)	IS: No SS results MBL: SS results (< short implants)	LWT + Graft + RCM SC
			Control (*n* = 38 I)	SFE + Standard-length implants (≤ 10 mm)	97.4% (1 failed) (*n* = 38)	1 yr: 38 (1.43 ± 0.47) 5 yr: 37 (2.10 ± 0.52)		
8	Guljé et al. 2019 [45]	5 years	Test (*n* = 21 I)	Short implants (6 mm)	94.7% (1 failed) (*n* = 21)	1 yr: 21 (0.10 ± 0.20) 5 yr: 20 (0.12 ± 0.36)	IS: No SS results MBL: No SS results	LWT + Graft SC
			Control (*n* = 20 I)	SFE + Standard-length implants (11 mm)	100% (0 failed) (*n* = 20)	1 yr: 20 (0.04 ± 0.33) 5 yr: 20 (0.14 ± 0.63)		
9	Thoma et al. 2018 [13]	5 years	Test (*n* = 60 I)	Short implants (6 mm)	98.5% (1 failed) (*n* = 60)	5 yr: 55 (0.45 ± 0.79)	IS: No SS results MBL: No SS results	LWT + Graft + RCM SC
			Control (*n* = 64 I)	SFE + Standard-length implants (11–15 mm)	100% (0 failed) (*n* = 64)	5 yr: 56 (0.45 ± 0.91)		
10	Bolle et al. 2018 [16]	1 year	Test (*n* = 37 I)	Short implants (4 mm)	91.9% (3 failed) (*n* = 37)	1 yr: 34 (0.63 ± 0.15)	IS: No SS results MBL: No SS results	LWT + Graft + RCM SC and FPD
			Control (*n* = 41 I)	OSFE + Standard-length implants (10 mm)	82.9% (7 failed) (*n* = 41)	1 yr: 35 (0.72 ± 0.25)		
11	Bechara et al. 2017 [2]	3 years	Test (*n* = 45 I)	Short implants (6 mm)	100% (0 failed) (*n* = 45)	1 yr: 45 (0.146) 3 yr: 44 (0.201)	IS: No SS results MBL: SS results (< short implants)	LWT + Graft SC and FPD
			Control (*n* = 45 I)	SFE + Standard-length implants (≤ 10 mm)	95.6% (2 failed) (*n* = 45)	1 yr: 43 (0.201) 3 yr: 43 (0.273)		
12	Gastaldi et al. 2017 [4]	3 years	Test (*n* = 16 I)	Short implants (5–6 mm)	100% (0 failed) (*n* = 16)	1 yr: 16 (0.78 ± 0.16) 3 yr: 16 (0.96 ± 0.21)	IS: No SS results MBL: No SS results	OSFE/LWT + Graft + RCM SC
			Control (*n* = 18 I)	SFE + Standard-length implants (10 mm)	100% (0 failed) (*n* = 18)	1 yr: 18 (0.95 ± 0.24) 3 yr: 14 (1.15 ± 0.30)		
13	Shi et al. 2019 [22]	1 year	Test (*n* = 75 I)	Short implants (6 mm)	100% (0 failed) (*n* = 75)	1 yr: 74 (0.51 ± 0.23)	IS: No SS results MBL: No SS results	OSFE SC
			Control (*n* = 75 I)	OSFE + Standard-length implants (8 mm)	100% (0 failed) (*n* = 75)	1 yr: 70 (0.47 ± 0.43)		
			Control (*n* = 75 I)	OSFE + Standard-length implants (10 mm)	100% (0 failed) (*n* = 75)	1 yr: 73 (0.52 ± 0.26)		
14	Schincaglia et al. 2015 [9]	1 year	Test (*n* = 67 I)	Short implants (6 mm)	100% (0 failed) (*n* = 67)	1 yr: 65 (0.39 ± 0.62)	IS: No SS results MBL: No SS results	LWT + Graft + RCM SC
			Control (*n* = 70 I)	SFE + Standard-length implants (11–15 mm)	100% (0 failed) (*n* = 70)	1 yr: 69 (0.22 ± 0.32)		

*IS*, implant survival; *MBL*, marginal bone loss; *I*, implants; *SFE*, sinus floor elevation; *SS*, statistically significant; *OSFE*, osteotome-mediated sinus floor elevation; *SC*, single crown; *LWT*, lateral window technique; *RCM*, resorbable collagen membrane; *FPD*, fixed partial denture.

### Studies quality assessment and bias risk

**Fig. 2:**
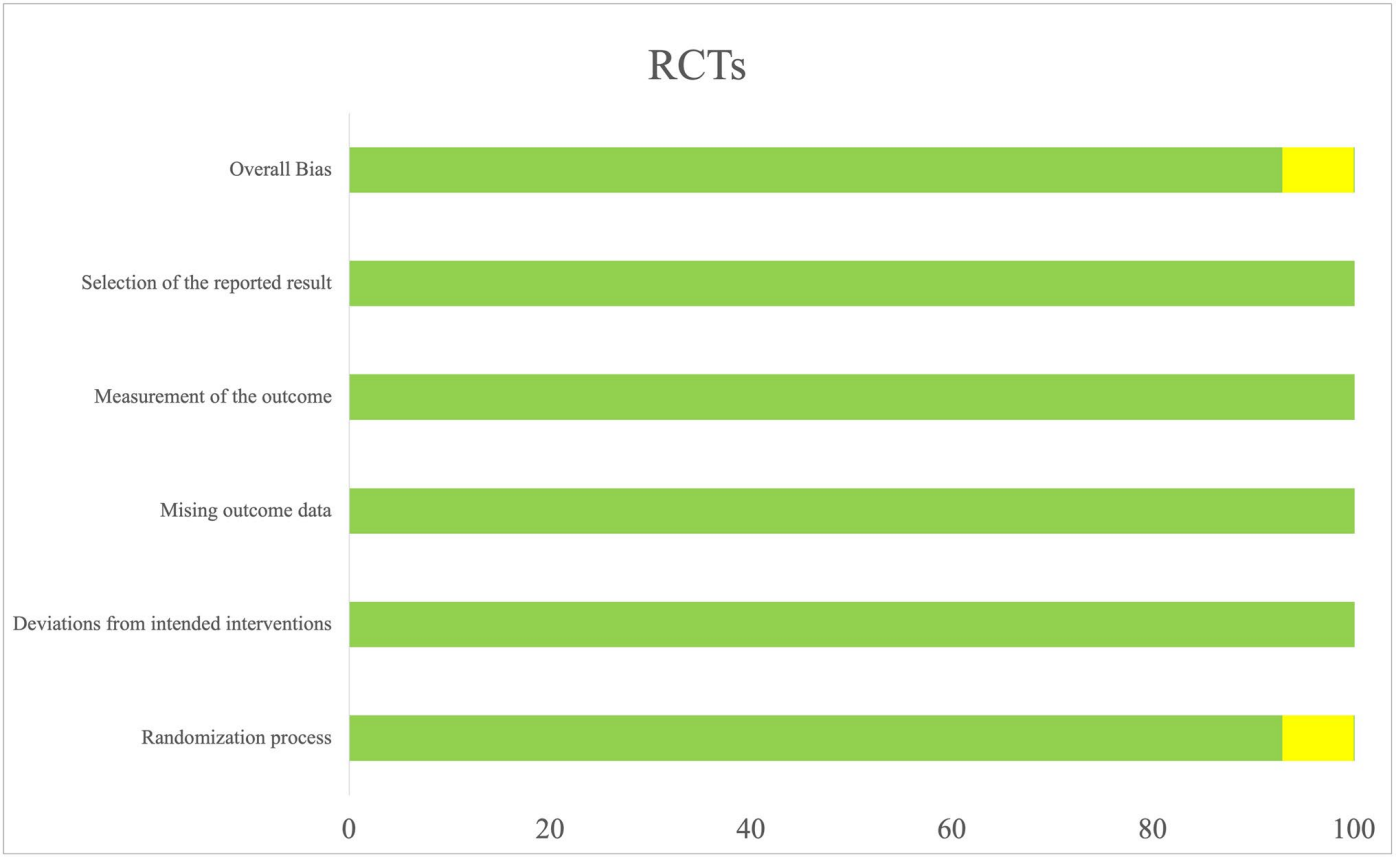
Assessing risk of bias in included studies by Robins-II Tool. The risk of bias of the included studies were judged as low (green), some concerns (yellow), or high (red)

The results of quality assessment and bias risk of the selected studies are summarized in Fig. 2. Most of the selected papers were considered as having low risk of bias.

### Primary and secondary outcomes

Fourteen studies (616 patients and 901 implants) examined both the IS and MBL. General characteristics of the included studies are displayed in Table 2.

**Fig. 3:**
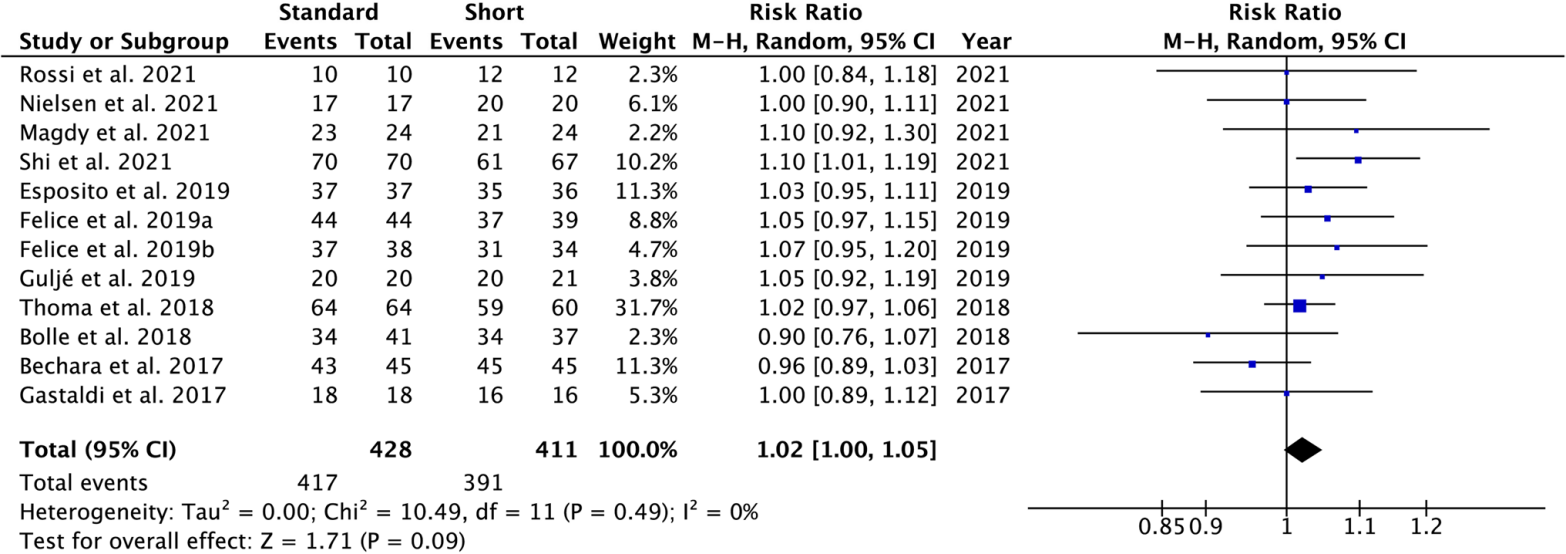
Forest plot for standard implants with sinus lift elevation (control group) versus short implants (test group) when comparing implant survival. Weighted mean is presented at CI 95%. Heterogeneity was determined using Higgins (*I*^2^). In all the analysis, a random-effects model was applied. Statistical significance was set at 0.05

**Fig. 4:**
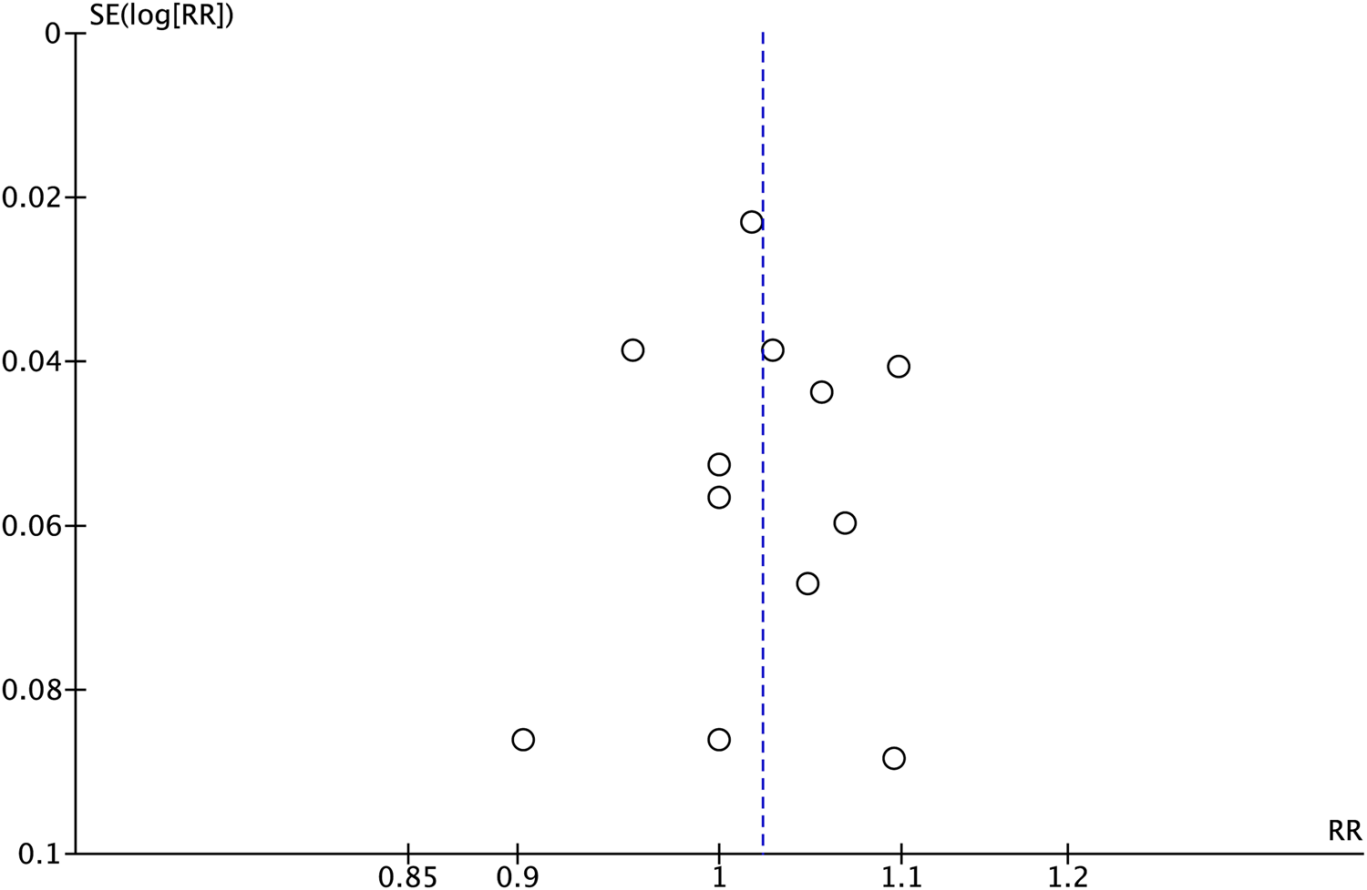
Funnel plot graph illustrating the publication bias and the systematic heterogeneity of the included studies. The standard error (SE) is represented in the vertical axis and the implant survival in the horizontal axis. The heterogeneity was considered low for the implant survival (*I*^2^ = 0%), so funnel plot did not show asymmetry, indicating the absence of publication bias

The IS, when comparing the experimental and control groups, was 1.02 (RR), ranging from 1.00 to 1.05 (CI 95%), suggesting that implant survival is similar when both techniques are used. Heterogeneity was low *I*^2^ = 0% and significance of the random-effect model was *p* = 0.09 (Fig. 3). IS forest plot graph is displayed in Fig. 3. Systematic heterogeneity is reflected at the funnel plot graph (Fig. 4).

**Fig. 5:**
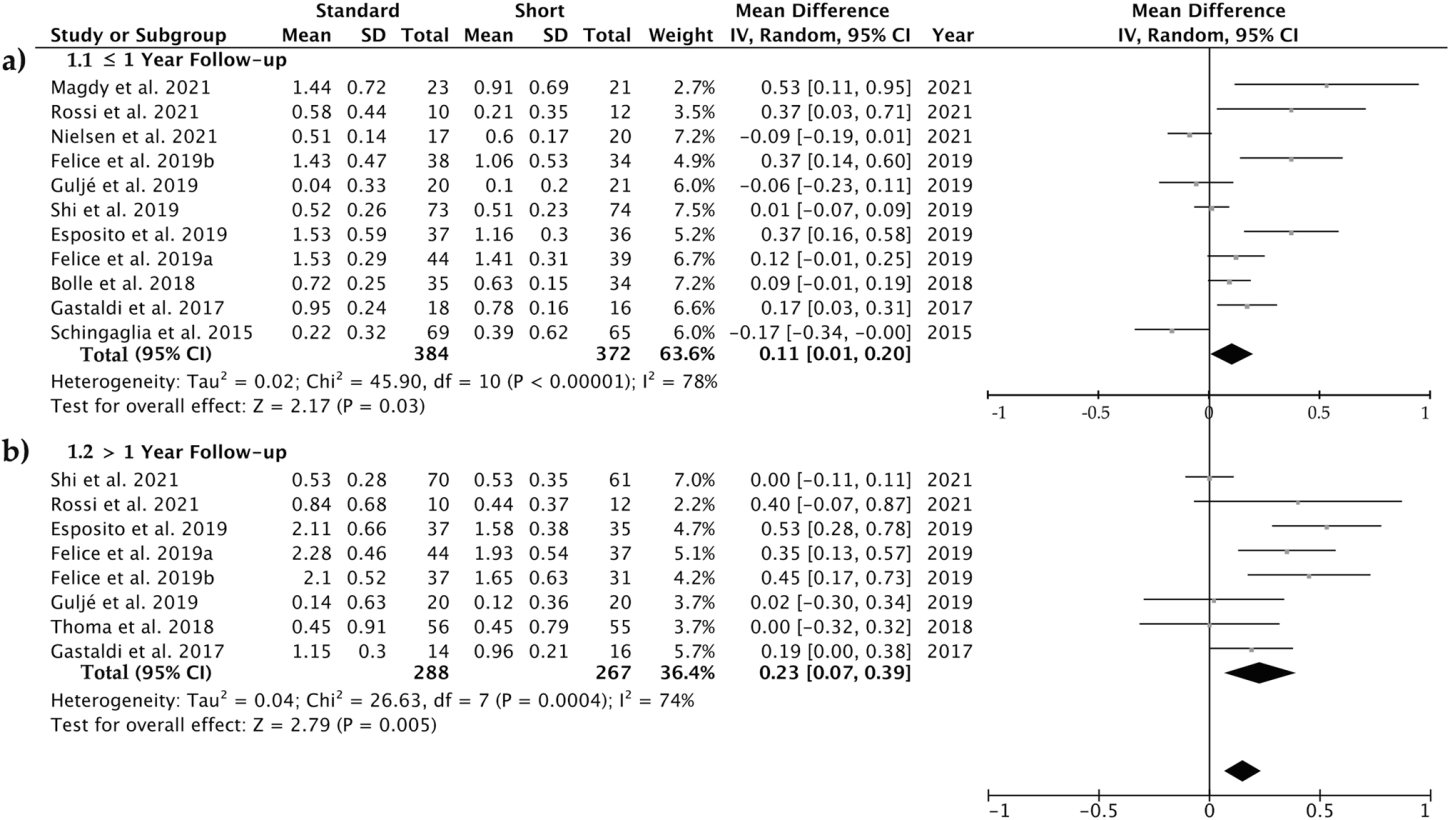
Forest plot for standard implants with sinus lift elevation (control group) versus short implants (test group) when comparing marginal bone loss. **a** One year of follow-up; **b** more than 1 year of follow-up. Weighted mean is presented at CI 95%. Heterogeneity was determined using Higgins (*I*^2^). In all the analysis, a random-effects model was applied. Statistical significance was set at 0.05

**Fig. 6:**
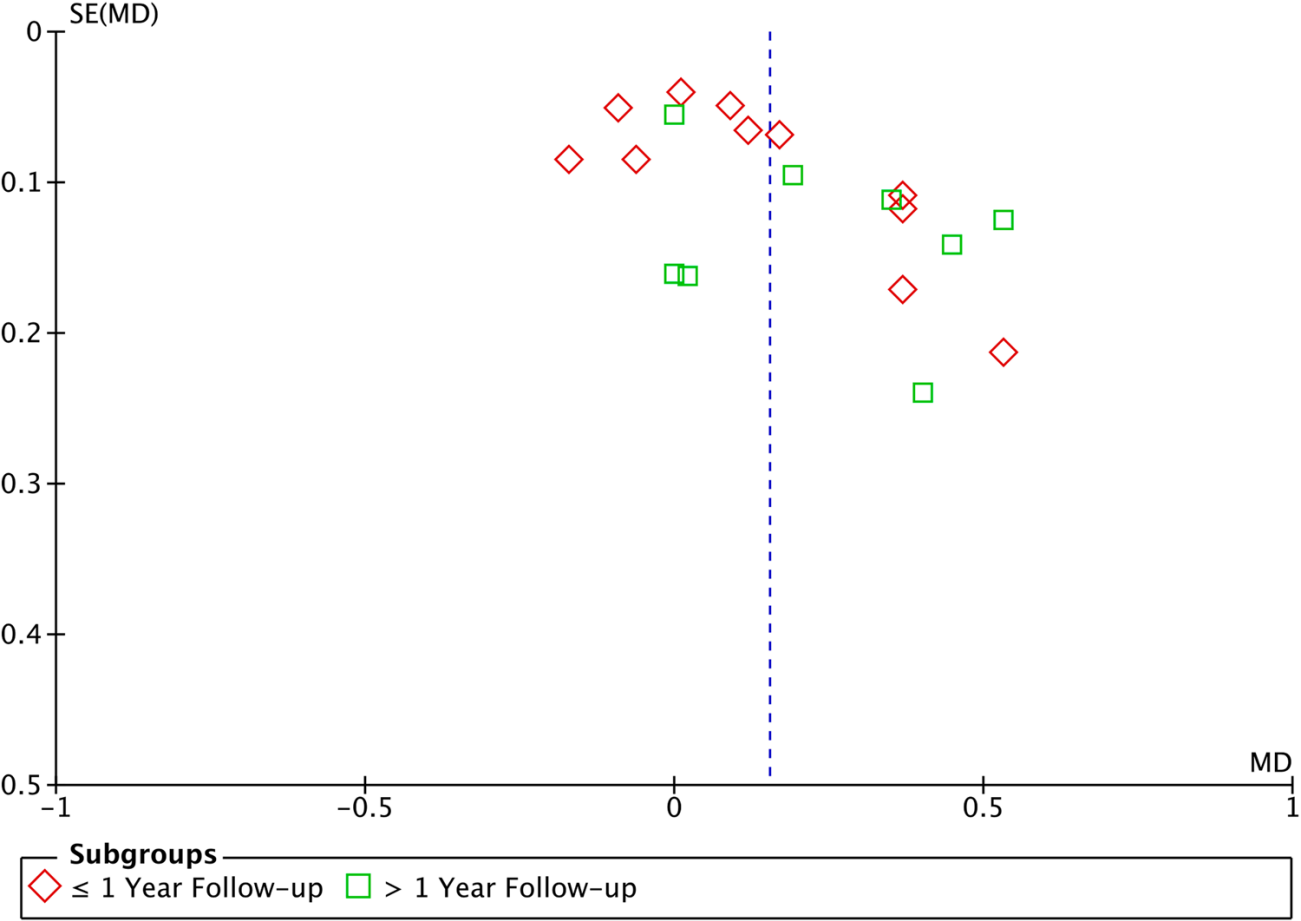
Funnel plot graph illustrating the publication bias and the systematic heterogeneity of the included studies. The standard error (SE) is represented in the vertical axis and the marginal bone loss in the horizontal axis. The heterogeneity was considered high for the marginal bone loss (*I*^2^ = 78% and *I*^2^ = 74%), so funnel plot shows asymmetry. The main reason of the asymmetry encountered could be due to the difference in the sample size of the included studies

The comparative studies performed in the two subgroups, a follow-up of less than or equal to 1 year and a follow-up of more than 1 year, show significant differences when the control and the test groups were compared. In the first subgroup, MBL was 0.11, ranging from 0.01 to 0.20 (CI 95%) (*p* = 0.03), indicating that the marginal bone loss is greater for standard implants with sinus lift elevation. Heterogeneity was high *I*^2^ = 78% and significance of the random-effects model was *p* = 0.03 (Fig. 5). After 1 year of follow-up, MBL was 0.23, ranging from 0.07 to 0.39 (CI 95%) (*p* = 0.005), indicating that the marginal bone loss is greater for standard-length implants with sinus lift elevation. Heterogeneity was high *I*^2^ = 74% and significance of the random-effects model was *p* = 0.005 (Fig. 5). MBL forest plot graph is displayed in Fig. 5. Systematic heterogeneity is displayed at the funnel plot graph (Fig. 6).

Secondary outcomes were also determined in the present research (Table 3). All papers reviewed in the present manuscript reported information regarding implant diameters. The most common diameter that was used corresponded to 4 mm [2, 5, 9, 13, 16, 45] and the less usual diameter was 3.3 mm [42]. Implant stability was analyzed in three studies [1, 2, 22]. Only Bechara et al. [2] reported significant differences at 3 years of follow-up.

**Table 3:** Summary of secondary outcomes collected from included studies

Author	Implant diameter *(mm)*	Implant surface	Implant stability (ISQ) *n* ± *SD*	BBT	BoP	PPD *(mm)*	Surgical time	Patient satisfaction	MUC/PI	Pain and swelling	Complications
Magdy et al. 2021 [1]	4.0/4.5/5	NR	No SS results	No SS results	NR	*Test*: SS results between 4 and 12 m follow-up *Control*: SS results between 4 and 12 m follow-up	NR	NR	NR	Pain: No SS results Swelling: SS results (> standard)	*Test*: 0 *Control*: 1
Shi et al. 2021 [42]	4.1/4.8	*SLA*: sandblasted and acid-etched	*Test*: T1: 69.6 ± 6.3 T2: 72.1 ± 5.7 *Control 1*: T1: 68 ± 5.4 T2: 71.9 ± 3.9 *Control 2*: T1: 71.8 ± 4.9 T2: 73.6 ± 5.2	NR	*Test*: 16.54% *Control 1*: 22.45% *Control 2*: 15.48% No SS results	*Test*: 3.03 ± 0.78 *Control 1*: 3.19 ± 0.67 *Control 2*: 3.04 ± 0.75 No SS results	Test < Control	Test > Control	MUC *Test*: 18 I *Control 1*: 21 I *Control 2*: 13 I PI *Test*: 2 I *Control 1*: 2 I *Control 2*: 1 I	NR	NR
Rossi et al. 2021 [43]	4.1	*SLA*: sandblasted and acid-etched	NR	NR	*Test*: 25% *Control*: 10% No SS results	*Test*: ≤ 4 *Control*: ≤ 4 No SS results	NR	Test > Control	NR	NR	NR
Nielsen et al. 2021 [3]	NR	*OsseoSpeed*: chemically modified with fluor	NR	NR	*Test*: 24% *Control*: 22% No SS results	*Test*: 2.4 ± 0.5 *Control*: 2.5 ± 0.6 No SS results	NR	NR	NR	Pain and swelling in 4 patients	Biological complication (> standard) SS results
Esposito et al. 2019 [10]	5	*Xpeed*: sandblasted and acid-etched (SLA) + calcium ions	NR	NR	NR	NR	NR	NR	MUC *Test*: 0 I *Control*: 1 I	NR	Membrane perforation and chipping prosthesis (> standard)
Felice et al. 2019 [5]	4	*SInergy*: blasting with alumina and cleaning with inert solvents	NR	NR	NR	NR	NR	Test > Control SS results	PI *Test*: 2 I *Control 1*: 0 I	NR	Membrane perforation and chipping prosthesis (> standard)
Felice et al. 2019 [44]	6	*RBM*: HA blasting and soft etching	NR	NR	NR	NR	NR	NR	PI *Test*: 1 I *Control*: 0 I	NR	Membrane perforation and decementation (> short)
Guljé et al. 2019 [45]	NR	*OsseoSpeed*: chemically modified with fluor	NR	NR	*Test*: 0% *Control*: 0% No SS results	*Test*: 2.8 ± 1.0 *Control*: 3.3 ± 0.8 No SS results	NR	*Test*: 9.4 ± 0.8 *Control*: 9.2 ± 0.8 No SS results	MUC *Test*: 22.2% *Control*: 47.42%	NR	*Test*: 4 *Control*: 1
Thoma et al. 2018 [13]	4	*OsseoSpeed*: chemically modified with fluor	NR	NR	*Test*: 40.9% *Control*: 50% No SS results	*Test*: 3.0 ± 1.0 *Control*: 3.0 ± 0.8 No SS results	NR	No SS results	MUC *Test*: 40.9% *Control*: 50% PI *Test*: 2% *Control*: 0%	NR	*Test*: 21 *Control*: 14
Bolle et al. 2018 [16]	4/4.5	*SA2*: sandblasting and etching	NR	NR	NR	NR	NR	NR	NR	NR	*Test*: 4 *Control*: 12
Bechara et al. 2017 [2]	4/4.5/5/ > 5	*Xpeed*: sandblasted and acid-etched (SLA) + calcium ions	*Test*: T1: 68.2 T3: 71.6 *Control*: T1: 67.8 T3: 72.4 SS results at T3	NR	NR	NR	Test < Control SS results	No SS results	NR	Pain and swelling in 14 patients (standard)	*Test*: 0 *Control*: 19 SS results
Gastaldi et al. 2017 [4]	5	*Osseotite*: dual acid-etching method	NR	NR	NR	NR	NR	NR	MUC *Test*: 0 I *Control*: 1 I	NR	*Test*: 2 *Control*: 1 No SS results
Shi et al. 2019 [22]	3.3/4.1/4.8	*SLA*: sandblasted and acid-etched	*Test*: T1: 69.6 ± 6.3 T2: 72.1 ± 5.7 *Control 1*: T1: 68 ± 5.4 T2: 71.9 ± 3.9 *Control 2*: T1: 71.8 ± 4.9 T2: 73.6 ± 5.2	NR	*Test*: 14.2% *Control 1*: 23.2% *Control 2*: 19.2% No SS results	*Test*: 3.0 ± 0.57 *Control 1*: 2.9 ± 0.50 *Control 2*: 3.0 ± 0.59 No SS results	*Test*: 13.6 ± 2.2 *Control 1*: 19.4 ± 3.7 *Control 2*: 18.3 ± 4.3 SS results	Control < Test SS results: discomfort No SS results: general overview	NR	No SS results	NR
Schincaglia et al. 2015 [9]	4	*OsseoSpeed*: chemically modified with fluor	NR	NR	*Test*: 53% *Control*: 38% SS results	*Test*: 2.8 ± 0.9 *Control*: 2.3 ± 1.4 No SS results	NR	NR	NR	NR	NR

*ISQ*, implant stability quotient; *BBT*, buccal bone thickness; *BoP*, bleeding on probing; *MUC*, mucositis; *PI*, peri-implantitis; *NR*, not reported; *SS*, statistically significant; *PPD*, probing pocket depth; *ISQ 1*, ISQ implant placement; *ISQ 2*, ISQ impressions; *ISQ 3*, ISQ 3 years; *HA*, hydroxyapatite.

Three articles reported that the implant surface was sandblasted and acid-etched (*SLA*) [22, 42, 43]. Nielsen et al. [3], Schincaglia et al. [9], Guljé et al. [45], and Thoma et al. [13] treated chemically modified with fluor the surface of the implants (*OsseoSpeed®*). Hydroxyapatite (HA) blasting and soft etching (*Xpeed®*) was employed to modify the implant surface in Esposito et al. [10] and Bechara et al. [2]. Blasting with alumina and cleaning with inert solvents (*SInergy®*) was employed by Felice et al. [5]. Bolle et al. [16] used sandblasting and etching to treat the implant surface (*SA2*). One paper (Gastaldi et al. [4]) treated the implant surface with dual acid-etching (*Osseolite®*). No information concerning the treatment of the implant surface was provided in Magdy et al. [1].

Only one article [1] published information regarding buccal bone thickness without showing significant differences between groups. One out of the fourteen revised manuscripts showed significant differences between groups, 53% and 38% in the test and control groups, respectively, when bleeding on probing was assessed [9]. Surgical time required was almost twice longer when standard-length implants were compared with short implants in Schincaglia et al. [9]. When probing depth was measured, only Magdy et al. [1] found significant differences, but within each study group when different follow-up periods were compared. The level of patient satisfaction was significantly higher in those cases treated with short implants, as Felice et al. [5] and Shi et al. [22] stated. When comparing both techniques, mucositis and peri-implantitis were not different throughout the fourteen revised papers. Pain and swelling were reported in 14 out 53 patients in Bechara et al. [2], and swelling significantly appeared more frequently in the group of the standard-length implants, as published by Magdy et al. [1]. More complications were reported in patients with standard-length implants [1–3, 5].

## Discussion

This systematic review and meta-analysis was aimed to identify the most reliable scientific information in regard to the implant survival (IS) and the MBL comparing short implants (≤ 6 mm) to standard-length implants (≥ 8 mm) performing sinus lift techniques. Attained results support that short dental implants (≤ 6 mm) promoted less MBL than standard-length dental implants (> 6 mm) used in cases of posterior atrophic maxilla that required lateral sinus lifting (Figs. 5a and 5b). When comparing MBL, the funnel plot (Fig. 6) shows an asymmetric distribution of the included studies, which tend to be placed in the upper side of the vertical axis. It is speculated that the lack of precision in studies with non-significant results may be the reason for this behavior. The average 0.11 mm (≤ 1 year of follow-up) and 0.23 mm (> 1 year of follow-up) of differences between groups was statistically significant, though it may have a slight clinical significance. Therefore, the null hypothesis must be partially accepted. Fourteen RCT studies comprised the present research. A total of 901 implants in 616 patients have been analyzed. Previously, similar objectives were proposed, but only ten RCTs with 775 patients [46] and seven RCTs involving 310 participants [20] were analyzed in both systematic review and meta-analysis, respectively. The control group, in our research, included three studies where OSFE and SC were performed. Bone graft was employed in studied patients of two papers. Bone graft and resorbable collagen membrane were used in patients analyzed of in nine of the tested papers.

Measurements of MBL have been utilized trying to analyze the long-term performance of dental implants [9], and it is a generally accepted parameter to assess the bone response around dental implants [47]. To guarantee long-term clinical service, the maintenance of a stable MBL becomes critical when short implants are used. Yan et al. [20] also obtained significantly less MBL at any follow-up than the control group. In addition, it has been previously reported that short implants could be a simpler, cheaper, and faster alternative inducing less morbidity when compared to standard-length implants placed after sinus elevation, if they could provide similar success rates [10].

One of the former studies with short implants reported 100% survival rate with no implant failures in the group of short implants, after 3 years of follow-up [2]. Patients lost an average of 1.02 mm of MBL around short implants and 1.54 mm around standard-length implants. A mean crestal bone loss of more than 1.5 mm after the first year of function and a MBL higher than 0.2 mm per year were considered as threshold values to determine implant success [9, 48]. In one of the analyzed papers [1], it has been recently reported that MBL is significantly lower in ultrashort (5.5 mm) implants comparing to standard-length implants (10 mm), after 12 months of follow-up period. However, in this study, three short implants failed, in contrast to the standard-length implants group, where only one implant failed (Table 2). Even shorter implants were analyzed by Esposito et al. [10] obtaining lower MBL (0.5 mm less in short implants). In this study, 5 × 5 mm (ultrashort) implants were placed and loaded after a follow-up of 5 years, only one failed of a test implant was reported (Table 2).

Any observed bone loss may be influenced by several factors in addition to the length of the implant, including implant’s geometry and design, surface configuration, crown fixation system, and surgical preparation [1]. Implants with a platform switching connection show significantly less MBL compared to implants with a butt joint connection [49]. Micro-threaded design in the most coronal aspect of the implant or extended to the neck of the implant leads to improved MBL. On the contrary, it has been stated that tissue level implants with smooth neck can lead to low peri-implant rates, though sandblasting plus acid-etched surface may also have influenced [22]. The fluoride-modified micro-rough implant surfaces may play a role in providing a stable MBL [9]. The machined surface (1.5 mm in ultrashort implants) [1] is advocated as one of the proposed causes for the decreased marginal bone loss [50]. Rough surface and wide diameter achieve higher bone-to-implant contact. Moreover, implant stress significantly raises with implant length [51]. It has been assumed that an increased crown-to-implant ratio might also create loading forces that could affect marginal bone stability [9]. Recently, it has been stated that a higher crown-to-implant ratio is not associated with increased risk of MBL [3]. Thereby, long-term study is needed to confirm the favorable design for predictability of short implants in the posterior maxilla. Another reason that can make the interpretation of results difficult is the fact that authors can consider the bone level at the implant placement as reference, instead of considering the bone level at the restoration placement [45]. Surgeon’s experience may also condition the clinical outcomes of the different treatment options [22]. Nevertheless, other papers revised in the present research did not find significant differences when both groups were compared within similar RCTs [4, 16, 22] (Table 2).

It is important to emphasize that a hypothetical bone loss of 2 mm around a 6 mm length is a clinical scenario which is not comparable to a 2 mm of bone loss around a 10–12 mm implant in terms of chance to re-create the lost tissues. In the first case, the bone-to-implant contact is relatively limited (one-third) in comparison with the second case (one-fifth). Even more, before assessing prognosis, several clinical factors should be considered such as, for instance, the individual susceptibility underlying the host response to the biofilm, the higher prevalence of biological complications, splinted or non-splinted implants, and data concerning oral hygiene. All these features are gathered in the Implant Disease Risk Assessment (IDRA) [52], where are referred the history of periodontitis, the percentage of tooth and implant sites with BOP, the number of tooth and implant sites with PD ≥ 5 mm, factors of radiographic bone loss in relation to age, the periodontitis susceptibility, the compliance of patients with supportive periodontal therapy, distance from the restorative margin to the bone crest, and factors related to the implant-supported prosthesis. Additionally, the shortcoming of determining the overall patient’s risk, not only the 2 mm bone loss, based only on the targeted 6-mm implant should be realized. In this aspect, the adhesion to an adequate maintenance care program has been shown to be crucial to preserve the obtained results in the long-term [53]. Moreover, further evidence about the impact of additional clinical aspects which were not included in the IDRA tool on the occurrence of biological complications and implant failure is required.

Regarding the survival rate of short implants, the present research has shown that implant length has no influence on implant survival, in concomitance with Yan et al. [20]; therefore, the null hypothesis must be partially rejected. It has been postulated that implant survival in short implants to be comparable to standard-length implants [54], though based on mid-term data, shorter dental implants rendered high implant survival rates [55] and less morbidity [13]. Nevertheless, contradictory outcomes may be found in the literature. On the one hand, it has been reported [46] that short implants exhibit lower predictability regarding survival rates when compared to longer implants (> 6 mm) after a follow-up period ranging between 1 and 5 years. Similarly, it has been stated that 10-mm implants combined with osteotome sinus floor elevation showed more favorable implant survival in comparison with short-6-mm implants [42]. In contrast, recent systematic reviews have reported that short and long dental implants have the same survival probability [1, 4, 13]. Several clinical studies have also confirmed similar survival rate between both groups [2, 9], tough Karthikeyan et al. [56] reported survival rates of 80–90% for implants ≤ 7 mm in a systematic review. The possible reasons for the inconsistency could be that the study population and implant systems varied in different clinical trials [42].

Concerning restoration (Table 2), if short implants were splinted or not should also be reported, as interconnected suprastructure does provide additional stability, influencing the clinical performance. Single units offer a more comfortable prosthetic approach, but transmitted more stress to restoration margins, whereas in splinted restorations, stress is mostly distributed to the implant neck [57]. Stress levels in the bone tissue surrounding splinted implants were markedly lower than stress levels surrounding uncoupled implants by a factor of nearly [51, 58]. In the present systematic review and meta-analysis, nine out fourteen papers used single crown for restoration. The implant survival, in this case, ranges from 100% [3, 4, 9, 22] until 87% [1]. Only one research utilized fixed partial denture, with a IS of 100% [43]. The rest (four manuscripts) placed single crowns and fixed partial dentures (Table 2), with a IS ranging from 97.2% [10] until 82.9% [16].

Implant diameters, in the present research, ranged from 3.3 [42] to 6 mm [5]. Differences in implant diameter introduced heterogeneity among studies with respect to MBL. Buccal bone thickness has been considered a secondary outcome in the clinical performance of implants, and it is usually measured during implant surgery at several distances from the implant shoulder [1]. BBT was higher in the standard-length group than in the short implants group at 12 months follow-up. Interestingly, ultrashort and standard-length implants exhibited an increase in buccal bone thickness at the 0-, 2-, and 4-mm level, when comparing baseline to the follow-ups [1]. Probing depth was measured by Magdy et al. [1] twice, from the gingival margin to the base of the peri-implant sulcus. When PD of short implants were analyzed, significant differences, mesially and distally, were obtained between 4- and 12-month follow-ups. Nevertheless, any significant difference between both groups appeared, denoting stability of the biological soft tissue seal around all implants [1]. The rest of the analyzed articles did not show significant differences in PD when compared, or data were not reported (Table 3).

Bleeding on probing showed a statistically significant difference between the groups with a higher number of subjects in the group of short implants. Shi et al. [42] reported 18 cases of mucositis and 2 of peri-implantitis in the test (short implants) group, and 34 cases of mucositis and 3 of peri-implantitis in the control (standard-length implants) group. No case of mucositis was found in the test group and only one in the control group, in Gastaldi et al. and Esposito et al. [4, 10]. By contrast, one [5] and two cases [44] of peri-implantitis were found in the test group and none in the control group. Mucositis was also assessed in Guljé et al. [45], who reported its presence in ⁓22% of short implants and ⁓47% of standard-length implants. Mucositis was diagnosed in around 50% of short and standard-length implants in Thoma et al. [13], and peri-implantitis was present in 2% of short implants and absent in standard-length implants [13].

Bechara et al. [2] found that, at 3 years, short implants showed a significantly higher mean implant stability quotient than the standard-length group (72.4 vs. 71.6). Nevertheless, implant stability measurements (mesiodistal and buccolingual) across the follow-ups showed no significant difference between the two treatment groups, confirming that the application of sinus elevation did not have any influence on implant stability regardless of the implant length [1, 8]. Pain scores, between treatment groups at all follow-up periods, were not statistically significant; swelling scores were higher in the standard-length group at 2, 3, and 5 days [1]. Swelling was also detected in 14 out of 45, in Bechara et al. [2], and in 4 patients out of 17, in Nielsen et al. [3], in both standard-length groups. Surgical time was significantly higher in the control group, ⁓32 min [2], than in the test group, ⁓19 min, as reported by previous studies [29, 30, 41] (Table 3). Shi et al. [22] described a ⁓30% lower surgical time with short implants than with standard implants. In general terms, the patient satisfaction was higher when short implants were used. Significantly less intra-operative discomfort was found in the patients with short implants, as Felice et al. [5] reported. This might indicate that short implants option resulted to be more attractive due to the high cost-effectiveness and patient satisfaction during the surgery, as published by some other previous studies [39, 41]. At a whole, complications, such as membrane perforation, mucositis, chipping prostheses, prostheses screws, and prostheses decementation, occurred more sparingly in patients treated with short implants [2, 3, 5, 10, 20, 44]. Implant migration into the sinus, often with the co-occurrence of sinus infection, has a higher prevalence in the elevation group [20]. Only one case in the standard-length group showed postoperative complications (benign paroxysmal positional vertigo), which improved within 6 weeks, in Magdy et al. [1]. Shorter implants are more prone for technical complications and should therefore be monitored more closely after loading [13], emphasizing in peri-implant health status and establishment of a balanced functional occlusion combined with a regular oral hygiene maintenance program [3]. Nevertheless, as a whole, the augmentation procedure is also far more technically demanding than placing short implants [36]. In general terms, the outcomes of the present study suggest that both treatments are viable treatment options that produce acceptable clinical and radiological outcomes. Short implants show the advantages of reduced postoperative discomfort, minimal invasiveness, reduced treatment time, and decreased cost [1].

One of the most remarkable limitations of the present study is the small sample size of the analyzed studies and the common short-term follow-up, 12 months in most of the studies [16], though some of them showed 5 years of follow-up period after loading [10, 44]. Hence, long-term studies are recommended to evaluate the short implants and the long-term prognosis, as reliable evidence on survival will depend on larger studies [1]*.* Limitations also include the difficulty to assess the risk of bias in several studies. The survival and success of short implants placed in severely resorbed jaws should not be compared with those of longer implants placed in adequate native bone but rather with the outcome of implants placed in grafted sites [51]. MBL has been calculated on panoramic radiographs. This represents a limit of the present study, as panoramic radiographs are per se subject to a certain degree of distortion. Cone beam computed tomography (CBCT), instead of 2D X-rays, could be a better way to evaluate the radiographic outcomes during the observation period [22]. Nevertheless, some authors [53] supported that both clinical and radiographic measurements did not follow a calibration session. They pointed out that data analysis did not allow generalizability to a population-based setting through a statistical examination [59]. Additional variables, such as patients’ oral hygiene habits, alcohol intake, periodontal status, and smoking status, should be considered for future studies. At present, only partially edentulous patients were included and a generalization of the results and recommendations for the use of shorter dental implants are limited to the present clinical indication. Nevertheless, the present study has several strengths: (*i*) it has been conducted a comprehensive literature search, and all included studies were RCTs, to accommodate the highest level of evidence and to add additional strength to the findings; (*ii*) subgroup analysis by follow-up length was performed to reduce bias across studies; and (*iii*) the risk of bias was low.

In order to get centered in the main goal of the present manuscript, a lack of reported biological complications comprising implant longevity associated to peri-implantitis [60] has been detected. Peri-implantitis has been defined as a plaque-associated pathological condition occurring in tissues around dental implants, characterized by inflammation in the peri-implant mucosa and subsequent progressive loss of supporting bone [59, 61] which can lead to the implant loss. It has been recommended [60] that before comparing biological complications of implants placed in native vs. augmented bone, the prevalence of peri-implantitis and the warnings of its interpretation should be discussed. Other biological complications such as the presence of titanium particles in the peri-implant soft tissues should also be addressed when some procedures, as implantoplasty, are going to be implemented [62].

One of the main limitations of the present systematic review and meta-analysis has been the incomplete information obtained about implant diameters, designs, type of bone grafts, and other secondary outcomes, leading to the impossibility of creating subgroups which would increase and complete our data analysis.

## Conclusions

Within the limitations of the present study, it can be concluded that short dental implants can be used as an alternative to standard-length implants plus sinus elevation, to support fixed prostheses in the rehabilitation of patients with an atrophic posterior maxilla. Higher marginal bone was observed in the groups of standard-length implants, but implant survival was similar in both groups. When short implants were used, a reduced postoperative discomfort, minimal invasiveness, shorter treatment time, and reduced costs were found. Further high-quality long-term studies are required to confirm these findings.

## Data Availability

The data presented in this study are available on request from the corresponding author.
